# Recent Insights into NCL Protein Function Using the Model Organism *Dictyostelium discoideum*

**DOI:** 10.3390/cells8020115

**Published:** 2019-02-02

**Authors:** Meagan D. McLaren, Sabateeshan Mathavarajah, Robert J. Huber

**Affiliations:** Department of Biology, Trent University, 1600 West Bank Drive, Peterborough, ON K9L 0G2, Canada; meaganmclaren@trentu.ca (M.D.M.); smathavarajah@trentu.ca (S.M.)

**Keywords:** Batten disease, neuronal ceroid lipofuscinosis, *Dictyostelium discoideum*, TPP1/CLN2, CLN3, CLN5, development

## Abstract

The neuronal ceroid lipofuscinoses (NCLs) are a group of devastating neurological disorders that have a global distribution and affect people of all ages. Commonly known as Batten disease, this form of neurodegeneration is linked to mutations in 13 genetically distinct genes. The precise mechanisms underlying the disease are unknown, in large part due to our poor understanding of the functions of NCL proteins. The social amoeba *Dictyostelium discoideum* has proven to be an exceptional model organism for studying a wide range of neurological disorders, including the NCLs. The *Dictyostelium* genome contains homologs of 11 of the 13 NCL genes. Its life cycle, comprised of both single-cell and multicellular phases, provides an excellent system for studying the effects of NCL gene deficiency on conserved cellular and developmental processes. In this review, we highlight recent advances in NCL research using *Dictyostelium* as a biomedical model.

## 1. Neuronal Ceroid Lipofuscinosis

The neuronal ceroid lipofuscinoses (NCLs), collectively known as Batten disease, are forms of neurodegeneration that affect people of all ages and ethnic backgrounds [1]. The pathological hallmark of the disease is the accumulation of autofluorescent storage bodies in almost every cell type and organ [2]. Storage body accumulation is caused by lysosomal dysfunction, which gradually leads to vision loss, epileptic seizures, impaired cognitive and motor function, and premature death [2,3]. Mutations in any one of 13 genetically distinct genes can cause Batten disease (*CLN1-8*, *CLN10-14*) [1]. These genes encode lysosomal enzymes (PPT1/CLN1, TPP1/CLN2, CLN5, CTSD/CLN10, CTSF/CLN13), proteins that peripherally associate with membranes (DNAJC5/CLN4, KCTD7/CLN14), proteins that are present in the secretory pathway (CLN5, PGRN/CLN11), and several transmembrane domain-containing proteins (CLN3, CLN6, MFSD8/CLN7, CLN8, ATP13A2/CLN12) [4]. The mechanisms underlying Batten disease are not well understood as the physiological functions of these proteins have not been fully established. 

## 2. Studying the Functions of NCL Proteins Using the Model Organism *Dictyostelium Discoideum*

Various genetic models have been used to study the functions of NCL proteins [5]. One such organism is the eukaryotic microbe *Dictyostelium discoideum*, which is firmly established as a model system for biomedical and human disease research [6,7]. Its 34 Mb haploid genome is fully sequenced, annotated, and encodes approximately 12,500 proteins [8]. The 24-h life cycle of *Dictyostelium* is comprised of distinct single-cell and multicellular phases, which allows for the study of conserved cellular and developmental processes [9]. Moreover, the ability to knockout genes using homologous recombination or CRISPR/Cas9-mediated targeting has made *Dictyostelium* a powerful model system for studying the functions of proteins linked to human disease [10,11]. 

**Figure 1:**
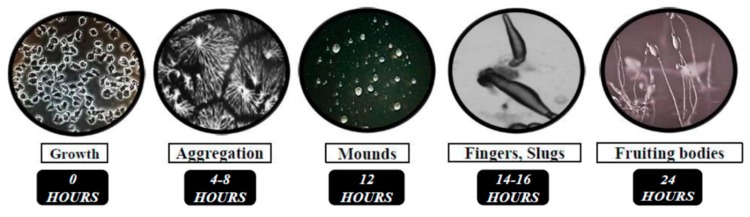
The 24-h life cycle of *Dictyostelium discoideum*. In nutrient-rich conditions, *Dictyostelium* grow as single cells and feed on readily available nutrients and bacteria. Removal of the food source initiates multicellular development. During the initial stages of development, cells chemotactically aggregate towards 3′,5′-cyclic adenosine monophosphate (cAMP) to form multicellular mounds. Cells then undergo a series of structural changes to form a finger followed by a motile pseudoplasmodium, or slug. Finally, the majority of cells within the slug terminally differentiate to form either stalk cells or spores in a fruiting body. Spores are dispersed and then germinate when nutrients become available, restarting the life cycle.

In nutrient-rich conditions, *Dictyostelium* grows as single cells, multiplying by mitosis and obtaining nutrients through endocytosis (Figure 1) [9]. Removal of nutrients prompts a 24-h developmental program consisting of a sequence of well-defined events (Figure 1). Cells first undergo chemotactic aggregation towards 3′,5′-cyclic adenosine monophosphate (cAMP) to form multicellular mounds (Figure 1). Mounds then undergo a series of morphological changes to form fingers that fall on the surface to generate motile pseudoplasmodia, also known as slugs (Figure 1). Finally, the majority of cells within the slug terminally differentiate into either stalk cells or spores, forming a fruiting body that completes the life cycle (Figure 1). Spore are then dispersed and germinate in the presence of nutrients, restarting the life cycle.

Like metazoan cells, *Dictyostelium* growth and development relies on fundamental processes including cell movement, cell sorting, cell differentiation, intracellular trafficking, autophagy, and signal transduction [9]. As a result, uncharacterized genes or undefined biological pathways can be thoroughly studied in *Dictyostelium*, and the results of these studies can then be translated to mammalian systems [12,13,14]. Work in *Dictyostelium* has made valuable contributions to our understanding of the functions of proteins linked to human neurological disorders, including epilepsy, prion diseases, lissencephaly, Alzheimer’s disease, Parkinson’ disease, and Huntington’s disease [15,16,17,18,19,20]. In addition, *Dictyostelium* has proven to be an exceptional organism for studying the cellular and molecular mechanisms underlying Batten disease [7]. The *Dictyostelium* genome encodes homologs of 11 of the 13 NCL genes, which is more than other model organisms including yeast, *C. elegans*, and *D. melanogaster* [7]. Recent work on *Dictyostelium* has provided fresh new insight into the functions of TPP1/CLN2, CLN3, and CLN5. In this review, we highlight these discoveries and discuss how these new findings have enhanced our knowledge of NCL protein function in humans. 

## 3. Using *Dictyostelium* to Study CLN2 Disease

### 3.1. Human TPP1

**Figure 2:**
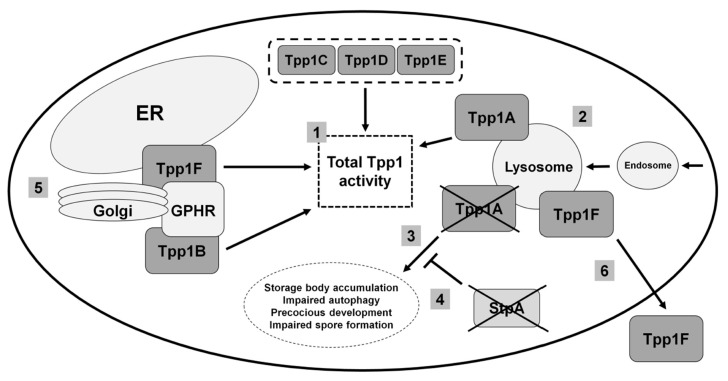
Current model of Tpp1 function in *Dictyostelium*. (1) The *Dictyostelium* genome encodes six proteins that show similarity to human TPP1/CLN2. These proteins likely all contribute to the total TPP1 activity in *Dictyostelium*. (2) Tpp1A and Tpp1F localize to the endocytic pathway including acidic compartments (e.g., lysosomes). (3) Loss of *tpp1A* causes storage body accumulation, impaired autophagy, precocious development, and impaired spore formation. (4) StpA functions as a second-site suppressor of *tpp1A*-deficiency in *Dictyostelium*. (5) Tpp1B and Tpp1F bind the Golgi pH regulator (GPHR). (5,6) Tpp1F also localizes to the endoplasmic reticulum (ER) and extracellular space.

Mutations in tripeptidyl peptidase 1 (*TPP1*) cause a late infantile form of NCL referred to as CLN2 disease [1]. Mutations in *TPP1/CLN2* are also linked to autosomal recessive spinocerebellar ataxia 7 (SCAR7) [21]. However, unlike in CLN2 disease where the activity of TPP1/CLN2 is completely abolished, the activity of the enzyme in SCAR7 patients is merely reduced [21]. As a result, SCAR7 patients do not exhibit vision loss or epilepsy [21]. TPP1/CLN2 is an acid-activated serine protease that localizes to the lysosomal matrix [22]. As a serine protease, TPP1/CLN2 is involved in several processes such as macroautophagy and endocytosis [23]. The study of TPP1/CLN2 in model organisms has been limited due to the absence of homologs in yeast, *C. elegans*, and *D. melanogaster* [7]. However, *Dictyostelium* has six genes that encode proteins that share a significant amount of similarity with human TPP1/CLN2 (genes: *tpp1A tpp1B*, *tpp1C*, *tpp1D*, *tpp1E*, and *tpp1F;* proteins: Tpp1A, Tpp1B, Tpp1C, Tpp1D, Tpp1E, and Tpp1F) [24,25] (Figure 2). 

### 3.2. Loss of the Lysosomal Enzyme Tpp1A Impairs Autophagy and Multicellular Development in Dictyostelium

Homologous recombination was used to knockout the *tpp1A* gene in *Dictyostelium* [24]. *tpp1A*-deficiency in *Dictyostelium* reduces overall Tpp1 activity and results in an accumulation of autofluorescent storage material in starved cells [24] (Figure 2). Like human TPP1/CLN2, Tpp1A localizes to the lysosome [22,24] (Figure 2). The growth and viability of *tpp1A^−^* cells is impaired in autophagy-stimulating media, which is consistent with previous work that reported reduced autophagosome formation in CLN2 disease patient fibroblasts [23,24] (Figure 2). During the mid-to-late stages of development, loss of *tpp1A* causes cells to develop precociously and form abnormal spores [24] (Figure 2). In addition, the development of *tpp1A^−^* cells is severely compromised in the presence of the lysosomotropic agent chloroquine, which is consistent with a role for Tpp1A at the lysosome [24]. By exploiting the genetic tractability of *Dictyostelium*, researchers used restriction enzyme-mediated integration (REMI) mutagenesis to identify *stpA* (suppressor of Tpp1 A) as a second site suppressor of *tpp1A*-deficiency [24] (Figure 2). StpA shares some similarity to oxysterol-binding proteins, which function in lipid transport and metabolism [24,26]. Intriguingly, altered lipid homeostasis has been linked to the NCLs [27,28]. For example, lipid accumulation has been observed in neural stem cells derived from induced pluripotent stem cells generated from CLN2 disease patient fibroblasts [27]. Thus, work in *Dictyostelium* has provided valuable new insight into the potential of targeting other genes that may reduce the effects of loss of function mutations in human *TPP1*. 

### 3.3. Tpp1B and Tpp1F Interact with the Golgi pH Regulator in Dictyostelium

As mentioned above, the *Dictyostelium* genome contains six genes that encode proteins similar to human TPP1/CLN2, with all six proteins likely contributing to the total TPP1 activity in the cell [24,25] (Figure 2). In addition to Tpp1A, recent work has also studied the function of Tpp1B and Tpp1F [25]. In *Dictyostelium*, both proteins bind the Golgi pH regulator (GPHR) [25] (Figure 2). GPHR is a transmembrane anion channel that acidifies compartments of the Golgi complex and influences its morphology as well as the morphology of the ER [29,30]. In *Dictyostelium*, the GPHR plays a role in regulating growth and the later stages of multicellular development [31]. In addition to the Golgi complex, Tpp1F localizes to the ER, V-ATPase-positive vesicles, and the extracellular space [25,32] (Figure 2). Like Tpp1A, Tpp1F also has serine protease activity [25]. However, *tpp1F*-deficiency in *Dictyostelium* has no obvious effects on growth or development, likely from the compensatory activities provided by the other Tpp1 proteins in *Dictyostelium* (expression of *tpp1B* is the highest during growth and development followed by *tpp1F* and *tpp1A*) [25,33]. In total, this work revealed a novel interaction of Tpp1 proteins with the GPHR in *Dictyostelium*, which should fuel research in mammalian models of CLN2 disease to determine if TPP1 interacts with the GPHR in human cells and how this interaction may contribute to the pathology underlying NCL.

## 4. Using *Dictyostelium* to Study CLN3 Disease

### 4.1. Human CLN3

Mutations in *CLN3* (ceroid lipofuscinosis neuronal 3) cause a juvenile form of NCL, which is the most common subtype of the disease [1]. *CLN3* encodes a 438-amino acid transmembrane protein that localizes to the late endosomal and lysosomal membranes [34,35]. Research in a diversity of genetic models has speculated that the function of CLN3 is linked to adhesion, apoptosis, autophagy, cell cycle control, cell proliferation, endocytosis, neurogenesis, osmoregulation, pH and ion homeostasis, and protein trafficking and secretion [32,36,37,38,39,40,41,42,43,44,45,46,47,48]. However, the precise function of the protein has not been defined. 

### 4.2. Loss of Cln3 Causes Pleiotropic Effects in Dictyostelium that are Consistent with its Localization to the Contractile Vacuole System

**Figure 3:**
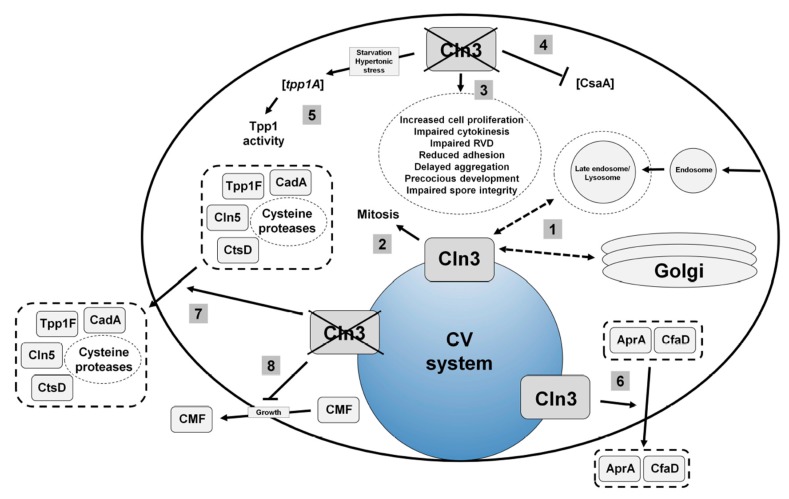
Current model of Cln3 function in *Dictyostelium*. (1) Cln3 localizes primarily to the contractile vacuole (CV) system, and to a smaller extent, compartments of the endocytic pathway and the Golgi complex. (2) Cln3 function is linked to mitosis. (3) Loss of *cln3* increases the rate of cell proliferation, alters cytokinesis, decreases the efficiency of regulatory volume decrease (RVD), reduces adhesion, delays aggregation, causes cells to develop precociously, and impairs spore integrity. (4) Loss of *cln3* reduces the intracellular level of CsaA protein. (5) Loss of *cln3* increases the expression of *tpp1A* during osmotic stress and increases TPP1 enzymatic activity. (6) Cln3 modulates the secretion of AprA and CfaD. (7) Loss of *cln3* increases the secretion of Tpp1F, Cln5, CtsD, CadA, and selected cysteine proteases. (8) Loss of *cln3* reduces the secretion of CMF during growth.

The *Dictyostelium* homolog of human CLN3 (gene: *cln3,* protein: Cln3) encodes a 421-amino acid transmembrane protein. In both growth and starved conditions, Cln3 localizes predominantly to the contractile vacuole (CV) system, and to a lesser extent, compartments of the endocytic pathway and Golgi complex [32,40,45] (Figure 3). During growth, *cln3^−^* cells display increased cell proliferation, aberrant cytokinesis, and defects in osmoregulation [40,48] (Figure 3). During multicellular development, *cln3^−^* cells display reduced cell-cell and cell-substrate adhesion, delayed aggregation, aberrant protein secretion, and precocious multicellular development [32,40,45] (Figure 3). Importantly, evidence from yeast and mammalian cell models also supports a role for CLN3 in these processes, highlighting that the molecular function of CLN3 is likely conserved from *Dictyostelium* to human [36,37,38,39,41,42,46,47]. 

The localization of Cln3 to the CV system has provided clues into the mechanism underlying *cln3*-deficiency phenotypes in *Dictyostelium*. The contractile vacuole (CV) system is a dynamic organelle that is linked to osmoregulation, protein secretion, and ion homeostasis [49,50,51]. The effect of *cln3*-deficiency on osmoregulation and protein secretion in *Dictyostelium* has been studied in detail and will be described below [32,48]. Previous work has also shown that *cln3*-deficiency phenotypes during development can be suppressed by treating cells with the calcium chelator egtazic acid (EGTA) [40,45]. These results are consistent with work showing aberrant calcium homeostasis in mouse models of CLN3 disease [43,46,52]. However, further work is needed to clarify the exact role of Cln3 in regulating ion balance in *Dictyostelium*. 

### 4.3. Cln3 Regulates Osmoregulation in Dictyostelium

During osmoregulation, the CV system regulates intracellular water balance by collecting excess water from the cytosol and then expelling the water out of the cell [49]. CLN3 has been shown to play a role in osmoregulation in mammalian models of CLN3 disease [37,38,39]. In a baby hamster kidney cell line, osmotic stress affects the expression and localization of CLN3 [38]. In mice, CLN3 regulates renal control of water and potassium balance [37]. Finally, osmotic stress induces an abnormal blood–brain barrier response in brain endothelial cells obtained from *Cln3*-deficient mice [39]. During hypotonic stress, the expulsion of water from the cytosol is known as regulatory volume decrease (RVD), which is a conserved process in all eukaryotic cells [53]. In *Dictyostelium*, previous work showed that *cln3^−^* cells display defects in RVD and exhibit a delay in their ability to recover from hypotonic stress [48] (Figure 3). This delay is exacerbated when *cln3^−^* cells are treated with ammonium chloride, a lysosomotropic compound that elevates the pH of intracellular compartments [48,54]. More specifically, following hypotonic stress, the ability of *cln3^−^* cells and V-ATPase-positive compartments in *cln3^−^* cells to reduce in size is delayed compared to wild-type cells [48]. The sensitivity of *cln3^−^* cells to hypotonic stress perpetuates into multicellular development where *cln3^−^* cells display delayed development under hypotonic stress, and arrested development at the slug stage when developed in hypotonic conditions with ammonium chloride [48]. These data suggest that lysosomotropic agents affect the ability of *cln3^−^* cells to cope with osmotic stress. In addition, *cln3^−^* cells display reduced viability under hypotonic stress, which also compromises the integrity of *cln3^−^* spores [48]. Finally, loss of *cln3* also impairs the viability and development of cells in response to hypertonic stress [48]. 

RNA sequencing was used to examine the pathways regulating the response of *cln3^−^* cells to osmotic stress. This analysis revealed 320 genes that were differentially expressed in *cln3^−^* cells compared to wild-type cells during hypotonic stress, and 162 genes that were differentially expressed during hypertonic stress [48]. The resulting datasets were then examined using GO term enrichment and STRING protein–protein interaction network analyses [55,56]. These analyses revealed that the differentially expressed genes are linked to developmental processes, which is consistent with the aberrant development of *cln3^−^* cells during osmotic stress [48]. Additionally, *cln3^−^* cells subjected to hypotonic stress displayed differential expression of genes linked to metabolic processes [48]. In both osmotic stress conditions, there was an enrichment of differentially expressed genes involved in transport and catalysis [48]. These results are consistent with the role of Cln3 in protein secretion, specifically the aberrant secretion of proteases by *cln3^−^* cells and the enhanced activity of Tpp1 in *cln3^−^* cells during hypertonic stress [32,48]. Finally, the proteins encoded by genes differentially expressed during hypotonic stress localize to the cell periphery and extracellular region, while proteins encoded by genes differentially expressed during hypertonic stress localize to membranes (e.g., intrinsic component of membrane) [48]. 

In *Dictyostelium*, GFP-Cln3 localizes to the CV system during mitosis and cytokinesis [48] (Figure 3). During cytokinesis, water efflux from CV system bladders facilitates the formation of the cleavage furrow, which is a transient structure that divides the two daughter cells [57,58,59]. Not surprisingly, *Dictyostelium* osmoregulatory mutants display defects in cytokinesis [60,61,62]. Aligning with the osmoregulatory defects observed in *cln3^−^* cells, loss of *cln3* increases the number of multi-nucleated cells in growth culture [48] (Figure 3). Importantly, these results are consistent with cytokinesis defects observed in a yeast model of CLN3 disease [36]. In total, this work links the function of Cln3 to osmoregulation in *Dictyostelium* and provides valuable new insight into the mechanisms underlying this function. 

### 4.4. Cln3 Regulates Protein Secretion in Dictyostelium

In addition to osmoregulation, the CV system has also been linked to protein secretion in *Dictyostelium* [50]. Work has shown that the enhanced proliferation of *cln3^−^* cells may be due to the aberrant secretion of proteins linked to growth, specifically autocrine proliferation repressor A (AprA) and counting factor-associated protein A (CfaD) [40] (Figure 3). AprA and CfaD function together to repress cell proliferation and facilitate chemorepulsion [63,64,65]. A preliminary analysis into the mechanism underlying the aberrant adhesion and aggregation of *cln3^−^* cells revealed that loss of *cln3* decreased the intracellular amount of the cell–cell adhesion protein contact site A (CsaA) and increased the amount of soluble extracellular calcium-dependent cell adhesion molecule A (CadA) [45] (Figure 3). The delayed aggregation of *cln3^−^* cells has also been linked to the reduced secretion of conditioned media factor (CMF) during growth [32] (Figure 3). Since CMF plays a critical role in initiating and synchronizing development upon starvation, these results indicate that *cln3^−^* cells may not be optimally primed to enter development [66]. 

Based on the above findings, mass spectrometry was used to further explore the effect of *cln3*-deficiency on protein secretion during aggregation [32]. That study provided the first evidence in any system showing that loss of *cln3* alters protein secretion [32]. A total of 450 proteins were detected in conditioned starvation buffer harvested from wild-type and *cln3^−^* cells [32]. Three proteins that are normally secreted by wild-type cells during starvation were absent in conditioned buffer harvested from *cln3^−^* cells [32]. Two of the three proteins function in adhesion and migration, which could explain the adhesion defects observed in *cln3^−^* cells [32,45]. In addition, 12 proteins that are not normally secreted during starvation were present in conditioned buffer harvested from *cln3^−^* cells [32]. Consistent with these findings, label-free quantification identified 42 proteins that were present in significantly higher amounts in *cln3^−^* conditioned starvation buffer compared to wild-type and 3 proteins that were present in significantly reduced amounts [32]. Gene ontology (GO) term analyses revealed an enrichment of proteins linked to endocytosis, vesicle-mediated transport, proteolysis, and metabolism. Importantly, these results support the reduced endocytosis and protein transport observed in cells from *Cln3*-deficient mice, reduced basal mitochondrial respiration and ATP production observed in mice harboring the most common mutation observed in patients with CLN3 disease, and the regulation of cathepsin D (CTSD) protease activity by CLN3 in baby hamster kidney cells [39,52,67]. In total, this work revealed for the first time that Cln3 plays a role in protein secretion and suggests that future research in *Dictyostelium* may provide additional insight on the precise role of CLN3 in regulating protein secretion in human cells. 

## 5. Using *Dictyostelium* to Study CLN5 Disease

### 5.1. Human CLN5

Mutations in *CLN5* (ceroid lipofuscinosis neuronal 5) cause a late-infantile form of Batten disease, but juvenile and adult onsets have also been reported [1,68,69]. CLN5 disease was first reported as a Finnish variant, however, patients with broad ethnic backgrounds have now been diagnosed [70,71,72,73]. In mammalian cells, CLN5 localizes to the lysosome and is present in the conditioned media of cultured cells, which is consistent with the presence of a signal peptide for secretion in the N-term of the protein [74,75,76,77]. CLN5 is first translated as a 407-amino acid type II transmembrane protein, which resides in the ER membrane [78]. The protein is then cleaved by signal peptide peptidase-like (SPPL) 3 to form a soluble protein [79,80]. In addition, CLN5 has eight N-glycosylation sites that are critical for the folding, trafficking, and localization of the protein [75]. Recently, human CLN5 was shown to display glycoside hydrolase activity [77]. CLN5 has also been speculated to function in autophagy, lipid metabolism, lysosome receptor sorting, myelination, and sphingolipid transport [81,82,83,84,85]. However, the precise mechanisms underlying CLN5 disease have yet to be revealed.

### 5.2. Cln5 is Secreted and Functions as a Glycoside Hydrolase in Dictyostelium

**Figure 4:**
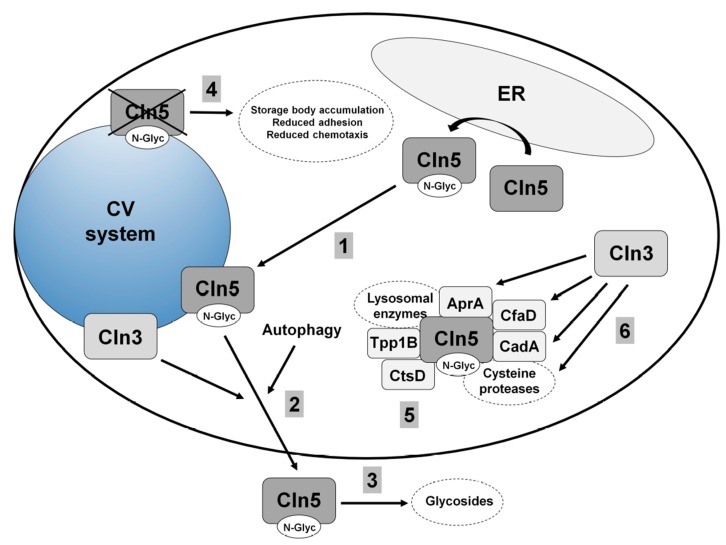
Current model of Cln5 function in *Dictyostelium*. (1) Cln5 is glycosylated in the endoplasmic reticulum (ER) and then trafficked to the contractile vacuole (CV) system. (2) Cln5 is secreted unconventionally via a pathway involving Cln3 and autophagy induction. (3) Cln5 functions as a glycoside hydrolase outside of the cell. (4) Loss of *cln5* leads to storage body accumulation and results in aberrant adhesion and chemotaxis. (5) Cln5 interacts with lysosomal enzymes, as well as the *Dictyostelium* homologs of human TPP1/CLN2, CTSD/CLN10, and CTSF/CLN13. (6) Cln5 interacts with proteins linked to Cln3 function in *Dictyostelium* (cysteine proteases, AprA, CfaD, and CadA).

*Dictyostelium* is one of the few early eukaryotes that contains a homolog of human CLN5 (yeast, *C. elegans*, and *D. melanogaster* lack a homolog) [7]. The *Dictyostelium* homolog (gene: *cln5*, protein: Cln5) is 322 amino acids in size, and like human CLN5, has glycoside hydrolase activity [77] (Figure 4). The first evidence for human CLN5 having glycoside hydrolase activity was based on studies that were initiated in *Dictyostelium* [77]. In *Dictyostelium*, Cln5 is glycosylated in the ER and then trafficked to the cell cortex where it appears to be secreted via the CV system during starvation [77,86] (Figure 4). Upon starvation in *Dictyostelium*, several conserved cellular processes are activated, one being autophagy, which is required for multicellular development [87]. Intriguingly, treatment of wild-type cells with lysosomotropic agents (e.g., ammonium chloride or chloroquine) decreases Cln5 secretion [86]. Since lysosomotropic compounds inhibit autophagy, these results suggest that autophagic mechanisms regulate the secretion of the protein [88] (Figure 4). In total, the secretion of Cln5 in *Dictyostelium* is consistent with observations in mammalian models of the disease and indicates that secreted CLN5 may play an important role in the pathological mechanisms underlying CLN5 disease. 

### 5.3. Loss of Cln5 Impairs Adhesion and Chemotaxis during the Early Stages of Dictyostelium Development

The accumulation of autofluorescent storage material in neurons, as well as cells outside the central nervous system, is a pathological hallmark of the NCLs [2]. In *Dictyostelium*, *cln5^−^* cells also accumulate autofluorescent storage deposits further highlighting the conserved nature of NCL pathways and validating the use of *Dictyostelium* as a model system for studying CLN5 disease [86] (Figure 4). During the early stages of multicellular development, *cln5^−^* cells display a reduced ability to adhere to the substrate, which is exacerbated when cells are treated with chloroquine [86]. Furthermore, *cln5^−^* cells also display a defect in cell–cell adhesion [86]. As a potential consequence of the aberrant adhesion, *cln5^−^* cells display reduced cAMP chemotaxis in a radial bioassay [86,89]. These results are consistent with observations of fibroblasts obtained from CLN5 disease patients, which attach poorly to tissue culture dishes and display altered expression of genes linked to cell adhesion [90]. Neurons from *Cln5*-deficient mice also display altered expression of genes linked to adhesion as well as aberrant localization of cytoskeletal proteins [91]. Finally, an analysis of the Cln5 interactome in *Dictyostelium* revealed that the protein interacts with lysosomal enzymes (e.g., alpha-mannosidase, beta-glucosidase), cysteine proteases, other NCL protein homologs such as Tpp1B, cathepsin D (CtsD), and uncharacterized protein DDB0252831 (which is similar to cathepsin F, CTSF/CLN13), and proteins linked to Cln3 function (e.g., AprA, CfaD, CadA) [77] (Figure 4). Therefore, future work in *Dictyostelium* may provide novel insight into the cellular pathways regulated by Cln5 and this knowledge can then be translated to other genetic models of CLN5 disease. 

## 6. Using *Dictyostelium* to Study the Molecular Networking of NCL Proteins

Mounting evidence indicates that the NCL proteins function in shared or convergent biological pathways [92]. Mutations in NCL proteins cause the accumulation of ceroid lipofuscin within cells and result in nearly identical clinical manifestations [2]. In addition, previous work reported the spatial and temporal co-expression of *TPP1/CLN2*, *CLN3*, and *CLN5* during brain development, shared interaction partners between CLN3 and CLN5, CLN5 polypeptides interacting directly with TPP1/CLN2 and CLN3, exacerbated NCL phenotypes in *Cln1*/*Cln5* double knockout mice, and the interaction of CLN5 with PPT1/CLN1, TPP1/CLN2, CLN3, CLN6, and CLN8 [41,93,94,95,96]. A recent report also showed decreased levels of CLN5 protein in a cell line derived from a *Mfsd8*/*Cln7* knockout mouse [97]. Thus, studying the function of any one NCL protein is likely to enhance our knowledge of the mechanisms underlying the neurodegeneration associated with the disease, knowledge that can then be applied to all subtypes of the disease.

Like mammalian models of Batten disease, there is evidence in *Dictyostelium* to support that the NCL proteins are connected at the molecular level. Using a proteomics-based approach, 10 of the 11 NCL protein homologs in *Dictyostelium* were detected in the macropinocytic pathway [98]. As discussed above, previous work revealed a function for Cln3 in protein secretion [32] (Figure 3). Specifically, that study reported that a loss of *cln3* increased the amount of Tpp1F, Cln5, and CtsD in conditioned starvation buffer [32] (Figure 2, Figure 3 and Figure 4). A follow-up study provided direct evidence linking the secretion of Cln5 to Cln3 function by showing increased amounts of Cln5 in conditioned starvation buffer harvested from *cln3^−^* cells [86] (Figure 3 and Figure 4). Furthermore, Cln5 was shown to co-localize with Cln3 at the CV system, which has been proposed to mediate its secretion [86]. Immunoprecipitation coupled with mass spectrometry revealed the Cln5 interactome in *Dictyostelium* [77]. Cln5-interactors include Tpp1B, CtsD, and uncharacterized protein DDB0252831 (similar to CTSF/CLN13), as well as proteins linked to Cln3 function in *Dictyostelium* (e.g., AprA, CfaD, CadA) [77]. Intriguingly, ten Cln5-interactors are differentially secreted by *cln3^−^* cells [32,77] (Table 1). Furthermore, *cln3*-deficiency increases the expression of *tpp1A* during hypertonic stress, which correlates with increased Tpp1 activity [48]. Finally, loss of *tpp1A*, *cln3*, or *cln5* in *Dictyostelium* causes similar phenotypes (Table 2). In total, these findings support the use of *Dictyostelium* to study the molecular networking of NCL proteins. 

## 7. Conclusions

*Dictyostelium* has proven to be an exceptional organism for studying the cellular roles of NCL proteins. Phenotypes previously revealed in other genetic models of Batten disease are present in *Dictyostelium* (e.g., aberrant autophagy, impaired osmoregulation, etc.) providing evidence that the functions of NCL proteins are likely conserved from *Dictyostelium* to human. Work in *Dictyostelium* has also revealed previously unknown functions for the NCL proteins, such as the role of CLN3 in protein secretion and the glycoside hydrolase activity of CLN5. These findings should spur future research in mammalian models of NCL to further explore these functions. In fact, recent studies in mice and humans have also linked the function of CLN3 to secretion [46,47]. However, as with any model organism, there are caveats that must be considered. For one, *Dictyostelium* has a limited number of cell types that may limit the translation of findings to specific tissues or organs in mammalian systems. In addition, since *Dictyostelium* lacks a nervous system, discoveries made in the organism must be validated in the relevant mammalian cell type. Nonetheless, *Dictyostelium* presents many benefits as a biomedical model system. Moving forward, research in *Dictyostelium* has the potential to identify molecular targets for therapies, which includes studying the effects of new drugs in a multicellular organism. Finally, NCL phenotypes overlap with those seen in patients with Alzhemier’s disease, Parkinson’s disease, and frontotemporal dementia [99,100,101]. Thus, on a larger scale, using *Dictyosteium* to study the functions of NCL proteins could enhance our understanding of the mechanisms underlying other forms of neurodegeneration. 

## Figures and Tables

**Table 1:** Proteins present in Cln5-GFP IP fractions that are differentially secreted by *cln3^−^* cells.

dictyBase ID	Protein Names	Gene Names
DDB0231036	Autocrine proliferation repressor protein A (PhoPQ-activated pathogenicity-related protein)	*aprA* DDB_G0281663
DDB0215012	Cathepsin D (Ddp44)	*ctsD*, *catD* DDB_G0279411
DDB0214999	Cysteine proteinase 4	*cprD*, *CP4* DDB_G0278721
DDB0185092	Cysteine proteinase 5	*cprE*, *CP5* DDB_G0272815
DDB0215005	Cysteine proteinase 7	*cprG*, *CP7* DDB_G0279187
DDB0191134	Elongation factor 1-alpha (EF-1-alpha) (50 kDa actin-binding protein) (ABP-50)	*eef1a2*, *efaa2*, *efaAII* DDB_G0269136
DDB0233663	Luminal-binding protein (BiP 2)	*bip2* DDB_G0276445
DDB0349243	Uncharacterized protein	DDB_G0288563
DDB0233868	Uncharacterized protein, member of the peptidase S28 family of serine proteases, a group containing lysosomal Pro-X carboxypeptidase, dipeptidyl-peptidase II, and thymus-specific serine peptidase	DDB_G0289749
DDB0238155	Induced after Legionella infection Contains a putative N-terminal signal sequence; regulated by gskA and zakA; induced by Legionella pneumophila infection	*iliA* DDB_G0285615

**Table 2:** Comparison of the phenotypes observed in *Dictyostelium* models of TPP1/CLN2, CLN3, and CLN5 disease.

Phenotype	*Tpp1a^−^*	*Cln3^−^*	*Cln5^−^*
Increased cell proliferation	No	Yes	Not known
Impaired cytokinesis	Not known	Yes	Not known
Autofluorescent inclusions	Yes	Not known	Yes
Defects in osmoregulation	Not known	Yes	Not known
Aberrant protein secretion	Not known	Yes	Not known
Reduced adhesion	Not known	Yes	Yes
Function linked to autophagy	Yes	Not known	Yes
Precocious development	Yes	Yes	Not known
Impaired spore formation	Yes	Not known	Not known
Reduced spore viability/integrity	No	Yes	Not known

## Abbreviations

AprA	autocrine proliferation repressor A
CadA	cell adhesion molecule A
cAMP	3′,5′-cyclic adenosine monophosphate
CfaD	counting factor-associated protein D
CLN	ceroid lipofuscinosis neuronal
CMF	conditioned media factor
CsaA	contact site A
CtsD	cathepsin D
CV	contractile vacuole
ER	endoplasmic reticulum
GPHR	Golgi pH regulator
NCL	neuronal ceroid lipofuscinosis
REMI	restriction enzyme-mediated integration
RVD	regulatory volume decrease
SCAR7	spinocerebellar ataxia 7
SPPL	signal peptide peptidase-like
StpA	suppressor of Tpp1 A
TPP1	tripeptidyl peptidase 1

## References

[1] Mole S.E., Cotman S.L. (2015). Genetics of the neuronal ceroid lipofuscinoses (Batten disease). Biochim. Biophys. Acta.

[2] Radke J., Stenzel W., Goebel H.H. (2015). Human NCL neuropathology. Biochim. Biophys. Acta.

[3] Schulz A., Kohlschütter A., Mink J., Simonati A., Williams R. (2013). NCL diseases—Clinical perspectives. Biochim. Biophys. Acta.

[4] Cárcel-Trullols J., Kovács A.D., Pearce D.A. (2015). Cell biology of the NCL proteins: What they do and don’t do. Biochim. Biophys. Acta.

[5] Bond M., Holthaus S., Tammen I., Tear G., Russell C. (2013). Use of model organisms for the study of neuronal ceroid lipofuscinosis. Biochim. Biophys. Acta.

[6] Müller-Taubenberger A., Kortholt A., Eichinger L. (2013). Simple system—Substantial share: The use of *Dictyostelium* in cell biology and molecular medicine. Eur. J. Cell Biol..

[7] Huber R.J. (2016). Using the social amoeba *Dictyostelium* to study the functions of proteins linked to neuronal ceroid lipofuscinosis. J. Biomed. Sci..

[8] Eichinger L., Pachebat J.A., Glöckner G., Rajandream M.-A., Sucgang R., Berriman M., Song J., Olsen R., Szafranski K., Xu Q. (2005). The genome of social amoeba *Dictyostelium discoideum*. Nature.

[9] Mathavarajah S., Flores A., Huber R.J. (2017). *Dictyostelium discoideum*: A model system for cell and developmental biology. Curr. Protoc. Essent. Lab. Tech..

[10] Faix J., Linkner J., Nordholz B., Platt J.L., Liao X.H., Kimmel A.R. (2013). The application of the Cre-loxP system for generating multiple knock-out and knock-in targeted loci. Methods Mol. Biol..

[11] Sekine R., Kawata T., Muramoto T. (2018). CRISPR/Cas9 mediated targeting of multiple genes in *Dictyostelium*. Sci. Rep..

[12] Terbach N., Shah R., Kelemen R., Klein P.S., Gordienko D., Brown N.A., Wilkinson C.J., Williams R.S. (2011). Identifying an uptake mechanism for the antiepileptic and bipolar disorder treatment valproic acid using the simple biomedical model *Dictyostelium*. J. Cell Sci..

[13] Chang P., Walker M.C., Williams R.S. (2014). Seizure-induced reduction in PIP3 levels contributes to seizure-activity and is rescued by valproic acid. Neurobiol. Dis..

[14] Alexander S., Alexander H. (2011). Lead genetic studies in *Dictyostelium discoideum* and translational studies in human cells demonstrate that sphingolipids are key regulators of sensitivity to cisplatin and other anticancer drugs. Semin. Cell Dev. Biol..

[15] Meyer I., Kuhnert O., Gräf R. (2011). Functional analyses of lissencephaly-related proteins in *Dictyostelium*. Semin. Cell Dev. Biol..

[16] Maniak M. (2011). *Dictyostelium* as a model for human lysosomal and trafficking diseases. Semin. Cell Dev. Biol..

[17] Myre M.A. (2012). Clues to γ-secretase, huntingtin and Hirano body normal function using the model organism *Dictyostelium discoideum*. J. Biomed. Sci..

[18] Walker M.C., Williams R.S. (2013). The search for better epilepsy treatments: From slime mould to coconuts. Biochem. Soc. Trans..

[19] Annesley S.J., Chen S., Francione L.M., Sanislav O., Chavan A.J., Farah C., De Piazza S.W., Storey C.L., Ilievska J., Fernando S.G. (2014). *Dictyostelium*, a microbial model for brain disease. Biochim. Biophys. Acta.

[20] Malinovska L., Alberti S. (2015). Protein misfolding in *Dictyostelium*: Using a freak of nature to gain insight into a universal problem. Prion.

[21] Sun Y., Almomani R., Breedveld G.J., Santen G.W., Aten E., Lefeber D.J., Hoff J.I., Brusse E., Verheijen F.W., Verdijk R.M. (2013). Autosomal recessive spinocerebellar ataxia 7 (SCAR7) is caused by variants in TPP1, the gene involved in classic late-infantile neuronal ceroid lipofuscinosis 2 disease (CLN2 disease). Hum. Mutat..

[22] Sleat D.E., Donnelly R.J., Lackland H., Liu C.G., Sohar I., Pullarkat R.K., Lobel P. (1997). Association of mutations in a lysosomal protein with classical late-infantile neuronal ceroid lipofuscinosis. Science.

[23] Vidal-Donet J.M., Cárcel-Trullols J., Casanova B., Aguado C., Knecht E. (2013). Alterations in ROS activity and lysosomal pH account for distinct patterns of macroautophagy in LINCL and JNCL fibroblasts. PLoS ONE.

[24] Phillips J.E., Gomer R.H. (2015). Partial genetic suppression of a loss-of-function mutant of the neuronal ceroid lipofuscinosis-associated protease TPP1 in *Dictyostelium discoideum*. Dis. Models Mech..

[25] Stumpf M., Müller R., Gaßen B., Wehrstedt R., Fey P., Karow M.A., Eichinger L., Glöckner G., Noegel A.A. (2017). A tripeptidyl peptidase 1 is a binding partner of the Golgi pH regulator (GPHR) in *Dictyostelium*. Dis. Models Mech..

[26] Olkkonen V.M., Li S. (2013). Oxysterol-binding proteins: Sterol and phosphoinositide sensors coordinating transport, signaling and metabolism. Prog. Lipid Res..

[27] Sima N., Li R., Huang W., Xu M., Beers J., Zou J., Titus S., Ottinger E.A., Marugan J.J., Xie X. (2018). Neural stem cells for disease modeling and evaluation of therapeutics for infantile (CLN1/PPT1) and late infantile (CLN2/TPP1) neuronal ceroid lipofuscinoses. Orphanet J. Rare Dis..

[28] Schultz M.L., Tecedor L., Lysenko E., Ramachandran S., Stein C.S., Davidson B.L. (2018). Modulating membrane fluidity corrects Batten disease phenotypes in vitro and in vivo. Neurobiol. Dis..

[29] Maeda Y., Ide T., Koike M., Uchiyama Y., Kinoshita T. (2008). GPHR is a novel anion channel critical for acidification and functions of the Golgi apparatus. Nat. Cell Biol..

[30] Charroux B., Royet J. (2014). Mutations in the *Drosophila* ortholog of the vertebrate Golgi pH regulator (GPHR) protein disturb endoplasmic reticulum and Golgi organization and affect systemic growth. Biol. Open.

[31] Deckstein J., van Appeldorn J., Tsangarides M., Yiannakou K., Müller R., Stumpf M., Sukumaran S.K., Eichinger L., Noegel A.A., Riyahi T.Y. (2015). The *Dictyostelium discoideum* GPHR ortholog is an endoplasmic reticulum and Golgi protein with roles during development. Eukaryot. Cell.

[32] Huber R.J. (2017). Loss of Cln3 impacts protein secretion in the social amoeba *Dictyostelium*. Cell. Signal..

[33] Rot G., Parikh A., Curk T., Kuspa A., Shaulsky G., Zupan B. (2009). dictyExpress: A *Dictyostelium discoideum* gene expression database with an explorative data analysis web-based interface. BMC Bioinform..

[34] Cotman S.L., Staropoli J.F. (2012). The juvenile Batten disease protein, CLN3, and its role in regulating anterograde and retrograde post-Golgi trafficking. Clin. Lipidol..

[35] Ratajczak E., Petcherski A., Ramos-Moreno J., Ruonala M.O. (2014). FRET-assisted determination of CLN3 membrane topology. PLoS ONE.

[36] Codlin S., Haines R.L., Burden J.J., Mole S.E. (2008). Btn1 affects cytokinesis and cell-wall deposition by independent mechanisms, one of which is linked to dysregulation of vacuole pH. J. Cell Sci..

[37] Stein C.S., Yancey P.H., Martins I., Sigmund R.D., Stokes J.B., Davidson B.L. (2010). Osmoregulation of ceroid neuronal lipofuscinosis type 3 in the renal medulla. Am. J. Physiol. Cell Physiol..

[38] Getty A., Kovács A.D., Lengyel-Nelson T., Cardillo A., Hof C., Chan C.H., Pearce D.A. (2013). Osmotic stress changes the expression and subcellular localization of the Batten disease protein CLN3. PLoS ONE.

[39] Tecedor L., Stein C.S., Schultz M.L., Farwanah H., Sandhoff K., Davidson B.L. (2013). CLN3 loss disturbs membrane microdomain properties and protein transport in brain endothelial cells. J. Neurosci..

[40] Huber R.J., Myre M.A., Cotman S.L. (2014). Loss of Cln3 function in the social amoeba *Dictyostelium discoideum* causes pleiotropic effects that are rescued by human CLN3. PLoS ONE.

[41] Fabritius A., Vesa J., Minye H.M., Nakano I., Kornblum H., Peltonen L. (2014). Neuronal ceroid lipofuscinosis genes, CLN2, CLN3 and CLN5 are spatially and temporally co-expressed in a developing mouse brain. Exp. Mol. Pathol..

[42] Mao D., Che J., Han S., Zhao H., Zhu Y., Zhu H. (2015). RNAi-mediated knockdown of the *CLN3* gene inhibits proliferation and promotes apoptosis in drug-resistant ovarian cancer cells. Mol. Med. Rep..

[43] Chandrachud U., Walker M.W., Simas A.M., Heetveld S., Petcherski A., Klein M., Oh H., Wolf P., Zhao W.N., Norton S. (2015). Unbiased cell-based screening in a neuronal cell model of Batten disease highlights an interaction between Ca^2+^ homeostasis, autophagy, and CLN3 protein function. J. Biol. Chem..

[44] Hong M., Song K.D., Lee H.K., Yi S., Lee Y.S., Heo T.H., Jun H.S., Kim S.J. (2016). Fibrates inhibit the apoptosis of Batten disease lymphoblast cells via autophagy recovery and regulation of mitochondrial membrane potential. In Vitro Cell. Dev. Biol. Anim..

[45] Huber R.J., Myre M.A., Cotman S.L. (2017). Aberrant adhesion impacts early development in a *Dictyostelium* model for juvenile neuronal ceroid lipofuscinosis. Cell Adhes. Migr..

[46] Parviainen L., Dihanich S., Anderson G.W., Wong A.M., Brooks H.R., Abeti R., Rezaie P., Lalli G., Pope S., Heales S.J. (2017). Glial cells are functionally impaired in juvenile neuronal ceroid lipofuscinosis and detrimental to neurons. Acta Neuropathol. Commun..

[47] Sleat D.E., Tannous A., Sohar I., Wiseman J.A., Zheng H., Qian M., Zhao C., Xin W., Barone R., Sims K.B. (2017). Proteomic analysis of brain and cerebrospinal fluid from the three major forms of neuronal ceroid lipofuscinosis reveals potential biomarkers. J. Proteome Res..

[48] Mathavarajah S., McLaren M.D., Huber R.J. (2018). Cln3 function is linked to osmoregulation in a *Dictyostelium* model of Batten disease. Biochim. Biophys. Acta.

[49] Du F., Edwards K., Shen Z., Sun B., De Lozanne A., Briggs S., Firtel R.A. (2008). Regulation of contractile vacuole formation and activity in *Dictyostelium*. EMBO J..

[50] Sriskanthadevan S., Brar S.K., Manoharan K., Siu C.H. (2013). Ca^2+^-calmodulin interacts with DdCAD-1 and promotes DdCAD-1 transport by contractile vacuoles in *Dictyostelium* cells. FEBS J..

[51] Plattner H. (2013). Contractile vacuole complex—Its expanding protein inventory. Int. Rev. Cell Mol. Biol..

[52] Bosch M.E., Kielian T. (2018). Astrocytes in juvenile neuronal ceroid lipofuscinosis (CLN3) display metabolic and calcium signaling abnormalities. J. Neurochem..

[53] Okada Y., Maeno E., Shimizu T., Dezaki K., Wang J., Morishima S. (2001). Receptor-mediated control of regulatory volume decrease (RVD) and apoptotic volume decrease (AVD). J. Physiol..

[54] Ascoli M., Puett D. (1978). Inhibition of the degradation of receptor-bound human choriogonadotropin by lysosomotropic agents, protease inhibitors, and metabolic inhibitors. J. Biol. Chem..

[55] Boyle E.I., Weng S., Gollub J., Jin H., Botstein D., Cherry J.M., Sherlock G. (2004). GO: TermFinder—Open source software for accessing Gene Ontology information and finding significantly enriched Gene Ontology terms associated with a list of genes. Bioinformatics.

[56] Szklarczyk D., Franceschini A., Wyder S., Forslund K., Heller D., Huerta-Cepas J., Simonovic M., Roth A., Santos A., Tsafou K.P. (2015). STRING v10: Protein-protein interaction networks, integrated over the tree of life. Nucleic Acids Res..

[57] Fukui Y., Inoué S. (1991). Cell division in *Dictyostelium* with special emphasis on actomyosin organization in cytokinesis. Cell Motil. Cytoskeleton.

[58] Zhu Q., Liu T., Clarke M. (1993). Calmodulin and the contractile vacuole complex in mitotic cells of *Dictyostelium discoideum*. J. Cell Sci..

[59] Neujahr R., Heizer C., Gerisch G. (1997). Myosin II-independent processes in mitotic cells of *Dictyostelium discoideum*: Redistribution of the nuclei, re-arrangement of the actin system and formation of the cleavage furrow. J. Cell Sci..

[60] Wienke D.C., Knetsch M.L., Neuhaus E.M., Reedy M.C., Manstein D.J. (1999). Disruption of a dynamin homologue affects endocytosis, organelle morphology, and cytokinesis in *Dictyostelium discoideum*. Mol. Biol. Cell.

[61] Rivero F., Illenberger D., Somesh B.P., Dislich H., Adam N., Meyer A.K. (2002). Defects in cytokinesis, actin reorganization and the contractile vacuole in cells deficient in RhoGDI. EMBO J..

[62] Gerald N.J., Siano M., De Lozanne A. (2002). The *Dictyostelium* LvsA protein is localized on the contractile vacuole and is required for osmoregulation. Traffic.

[63] Brock D.A., Gomer R.H. (2005). A secreted factor represses cell proliferation in *Dictyostelium*. Development.

[64] Bakthavatsalam D., Brock D.A., Nikravan N.N., Houston K.D., Hatton R.D., Gomer R.H. (2008). The secreted *Dictyostelium* protein CfaD is a chalone. J. Cell Sci..

[65] Phillips J.E., Gomer R.H. (2012). A secreted protein is an endogenous chemorepellant in *Dictyostelium discoideum*. Proc. Natl. Acad. Sci. USA.

[66] Gomer R.H., Yuen I.S., Firtel R.A. (1991). A secreted 80 × 10^3^ Mr protein mediates sensing of cell density and the onset of development in *Dictyostelium*. Development.

[67] Carcel-Trullols J., Kovacs A.D., Pearce D.A. (2017). Role of the lysosomal membrane protein, CLN3, in the regulation of cathepsin D activity. J. Cell. Biochem..

[68] Cannelli S., Nardocci N., Cassandrini D., Morbin M., Aiello C., Bugiani M., Criscuolo L., Zara F., Striano P., Granata T. (2007). Revelation of a novel CLN5 mutation in early juvenile neuronal ceroid lipofuscinosis. Neuropediatrics.

[69] Mancini C., Nassani S., Guo Y., Chen Y., Giorgio E., Brussino A., Di Gregorio E., Cavalieri S., Lo Buono N., Funaro A. (2015). Adult-onset autosomal recessive ataxia associated with neuronal ceroid lipofuscinosis type 5 gene (CLN5) mutations. J. Neurol..

[70] Savukoski M., Klockars T., Holmberg V., Santavuori P., Lander E.S., Peltonen L. (1998). CLN5, a novel gene encoding a putative transmembrane protein mutated in Finnish variant late infantile neuronal ceroid lipofuscinosis. Nat. Genet..

[71] Xin W., Mullen T.E., Kiely R., Min J., Feng X., Cao Y., O’Malley L., Shen Y., Chu-Shore C., Mole S.E. (2010). CLN5 mutations are frequent in juvenile and late-onset non-Finnish patients with NCL. Neurology.

[72] Santorelli F.M., Garavaglia B., Cardona F., Nardocci N., Bernardina B.D., Sartori S., Suppiej A., Bertini E., Claps D., Battini R. (2013). Molecular epidemiology of childhood neuronal ceroid-lipofuscinosis in Italy. Orphanet J. Rare Dis..

[73] Simonati A., Williams R.E., Nardocci N., Laine M., Battini R., Schulz A., Garavaglia B., Moro F., Pezzini F., Santorelli F.M. (2017). Phenotype and natural history of variant late infantile ceroid-lipofuscinosis 5. Dev. Med. Child Neurol..

[74] Isosomppi J., Vesa J., Jalanko A., Peltonen L. (2002). Lysosomal localization of the neuronal ceroid lipofuscinosis CLN5 protein. Hum. Mol. Genet..

[75] Moharir A., Peck S.H., Budden T., Lee S.Y. (2013). The role of N-glycosylation in folding, trafficking, and functionality of lysosomal protein CLN5. PLoS ONE.

[76] Hughes S.M., Hope K.M., Xu J.B., Mitchell N.L., Palmer D.N. (2014). Inhibition of storage pathology in prenatal CLN5-deficient sheep neural cultures by lentiviral gene therapy. Neurobiol. Dis..

[77] Huber R.J., Mathavarajah S. (2018). Cln5 is secreted and functions as a glycoside hydrolase in *Dictyostelium*. Cell. Signal..

[78] Larkin H., Ribeiro M.G., Lavoie C. (2013). Topology and membrane anchoring of the lysosomal storage disease-related protein CLN5. Hum. Mutat..

[79] De Silva B., Adams J., Lee S.Y. (2015). Proteolytic processing of the neuronal ceroid lipofuscinosis related lysosomal protein CLN5. Exp. Cell Res..

[80] Jules F., Sauvageau E., Dumaresq-doiron K., Mazzaferri J., Haug-Kröper M., Fluhrer R., Costantino S., Lefrancois S. (2017). CLN5 is cleaved by members of the SPP/SPPL family to produce a mature soluble protein. Exp. Cell Res..

[81] Mamo A., Jules F., Dumaresq-Doiron K., Costantino S., Lefrancois S. (2012). The role of ceroid lipofuscinosis neuronal protein 5 (CLN5) in endosomal sorting. Mol. Cell. Biol..

[82] Schmiedt M.L., Blom T., Blom T., Kopra O., Wong A., von Schantz-Fant C., Ikonen E., Kuronen M., Jauhiainen M., Cooper J.D. (2012). *Cln5*-deficiency in mice leads to microglial activation, defective myelination and changes in lipid metabolism. Neurobiol. Dis..

[83] Haddad S.E., Khoury M., Daoud M., Kantar R., Harati H., Mousallem T., Alzate O., Meyer B., Boustany R. (2012). CLN5 and CLN8 protein association with ceramide synthase: Biochemical and proteomic approaches. Electrophoresis.

[84] Leinonen H., Keksa-Goldsteine V., Ragauskas S., Kohlmann P., Singh Y., Savchenko E., Puranen J., Malm T., Kalesnykas G., Koistinaho J. (2017). Retinal degeneration in a mouse model of CLN5 disease is associated with compromised autophagy. Sci. Rep..

[85] Adams J., Feuerborn M., Molina J.A., Wilden A.R., Adhikari B., Budden T., Lee S.Y. (2019). Autophagy-lysosome pathway alterations and alpha-synuclein up-regulation in the subtype of neuronal ceroid lipofuscinosis, CLN5 disease. Sci. Rep..

[86] Huber R.J., Mathavarajah S. (2018). Secretion and function of Cln5 during the early stages of *Dictyostelium* development. Biochim. Biophys. Acta.

[87] Mesquita A., Cardenal-Muñoz E., Dominguez E., Muñoz-Braceras S., Nuñez-Corcuera B., Phillips B.A., Tábara L.C., Xiong Q., Coria R., Eichinger L. (2017). Autophagy in *Dictyostelium*: Mechanisms, regulation and disease in a simple biomedical model. Autophagy.

[88] Rote K.V., Rechsteiner M. (1983). Degradation of microinjected proteins: Effects of lysosomotropic agents and inhibitors of autophagy. J. Cell. Physiol..

[89] O’Day D.H. (1979). Aggregation during sexual development in *Dictyostelium discoideum*. Can. J. Microbiol..

[90] Schulz A., Dhar S., Rylova S., Dbaibo G., Alroy J., Hagel C., Artacho I., Kohlschütter A., Lin S., Boustany R.M. (2004). Impaired cell adhesion and apoptosis in a novel CLN9 Batten disease variant. Ann. Neurol..

[91] von Schantz C., Saharinen J., Kopra O., Cooper J.D., Gentile M., Hovatta I., Peltonen L., Jalanko A. (2008). Brain gene expression profiles of Cln1 and Cln5 deficient mice unravels common molecular pathways underlying neuronal degeneration in NCL diseases. BMC Genom..

[92] Persaud-Sawin D.A., Mousallem T., Wang C., Zucker A., Kominami E., Boustany R.M. (2007). Neuronal ceroid lipofuscinosis: A common pathway?. Pediatr. Res..

[93] Vesa J., Chin M.H., Oelgeschläger K., Isosomppi J., DellAngelica E.C., Jalanko A., Peltonen L. (2002). Neuronal ceroid lipofuscinoses are connected at molecular level: Interaction of CLN5 protein with CLN2 and CLN3. Mol. Biol. Cell.

[94] Lyly A., von Schantz C., Heine C., Schmiedt M.L., Sipilä T., Jalanko A., Kyttälä A. (2009). Novel interactions of CLN5 support molecular networking between neuronal ceroid lipofuscinosis proteins. BMC Cell Biol..

[95] Scifo E., Szwajda A., Dębski J., Uusi-Rauva K., Kesti T., Dadlez M., Gingras A.C., Tyynelä J., Baumann M.H., Jalanko A. (2013). Drafting the CLN3 protein interactome in SH-SY5Y human neuroblastoma cells: A label-free quantitative proteomics approach. J. Proteome Res..

[96] Blom T., Schmiedt M.L., Wong A.M., Kyttälä A., Soronen J., Jauhiainen M., Tyynelä J., Cooper J.D., Jalanko A. (2013). Exacerbated neuronal ceroid lipofuscinosis phenotype in Cln1/5 double-knockout mice. Dis. Models Mech..

[97] Danyukova T., Ariunbat K., Thelen M., Brocke-Ahmadinejad N., Mole S.E., Storch S. (2018). Loss of CLN7 results in depletion of soluble lysosomal proteins and impaired mTOR reactivation. Hum. Mol. Genet..

[98] Journet A., Klein G., Brugière S., Vandenbrouck Y., Chapel A., Kieffer S., Bruley C., Masselon C., Aubry L. (2012). Investigating the macropinocytic proteome of *Dictyostelium* amoebae by high-resolution mass spectrometry. Proteomics.

[99] Deng H., Xiu X., Jankovic J. (2015). Genetic convergence of Parkinson’s disease and lysosomal storage disorders. Mol. Neurobiol..

[100] Dearborn J.T., Harmon S.K., Fowler S.C., O’Malley K.L., Taylor G.T., Sands M.S., Wozniak D.F. (2015). Comprehensive functional characterization of murine infantile Batten disease including Parkinson-like behavior and dopaminergic markers. Sci. Rep..

[101] Qureshi Y.H., Patel V.M., Berman D.E., Kothiya M.J., Neufeld J.L., Vardarajan B., Tang M., Reyes-Dumeyer D., Lantigua R., Medrano M. (2018). An Alzheimer’s disease-linked loss-of-function CLN5 variant impairs cathepsin D maturation, consistent with a retromer trafficking defect. Mol. Cell. Biol..

